# Globally distributed bacteriophage genomes reveal mechanisms of tripartite phage–bacteria–coral interactions

**DOI:** 10.1093/ismejo/wrae132

**Published:** 2024-07-20

**Authors:** Bailey A Wallace, Natascha S Varona, Poppy J Hesketh-Best, Alexandra K Stiffler, Cynthia B Silveira

**Affiliations:** Department of Biology, University of Miami, Coral Gables, FL 33146, United States; Department of Biology, University of Miami, Coral Gables, FL 33146, United States; Department of Biology, University of Miami, Coral Gables, FL 33146, United States; Department Veterinary Population Medicine, University of Minnesota, St. Paul, MN 55108, United States; Department of Biology, University of Miami, Coral Gables, FL 33146, United States; Department of Biology, University of Miami, Coral Gables, FL 33146, United States; Department of Marine Biology and Ecology, Rosenstiel School of Marine, Atmospheric, and Earth Science, University of Miami, Miami, FL 33149, United States

**Keywords:** viruses, scleractinian corals, bacteria, metabolic genes, symbiosis

## Abstract

Reef-building corals depend on an intricate community of microorganisms for functioning and resilience. The infection of coral-associated bacteria by bacteriophages can modify bacterial ecological interactions, yet very little is known about phage functions in the holobiont. This gap stems from methodological limitations that have prevented the recovery of high-quality viral genomes and bacterial host assignment from coral samples. Here, we introduce a size fractionation approach that increased bacterial and viral recovery in coral metagenomes by 9-fold and 2-fold, respectively, and enabled the assembly and binning of bacterial and viral genomes at relatively low sequencing coverage. We combined these viral genomes with those derived from 677 publicly available metagenomes, viromes, and bacterial isolates from stony corals to build a global coral virus database of over 20,000 viral genomic sequences spanning four viral realms. The tailed bacteriophage families *Kyanoviridae* and *Autographiviridae* were the most abundant, replacing groups formerly referred to as *Myoviridae* and *Podoviridae*, respectively. Prophage and CRISPR spacer linkages between these viruses and 626 bacterial metagenome-assembled genomes and bacterial isolates showed that most viruses infected *Alphaproteobacteria*, the most abundant class, and less abundant taxa like *Halanaerobiia* and *Bacteroidia*. A host–phage–gene network identified keystone viruses with the genomic capacity to modulate bacterial metabolic pathways and direct molecular interactions with eukaryotic cells. This study reveals the genomic basis of nested symbioses between bacteriophage, bacteria, and the coral host and its endosymbiotic algae.

## Introduction

Microorganisms of the coral holobiont are fundamental to the ecological success of reef-building corals [1]. A complex network of symbiotic interactions connects all coral holobiont entities, including viruses, bacteria, archaea, fungi, and dinoflagellates, broadening the functional capacity of the coral host and influencing the way the coral adapts and interacts with the reef environment [2, 3]. In addition to the well-described coral–Symbiodiniaceae endosymbiosis, prokaryotic communities (bacteria and archaea) play crucial roles in coral health [4]. These assemblages remain flexible in response to a changing environment and can mitigate the effects of environmental stressors [5]. Though we have gained a significant understanding of many coral–bacteria interactions, little is known about the roles of viruses in the holobiont. Currently available metagenomic tools have shown that viruses, particularly double-stranded DNA viruses infecting prokaryotes (herein, phages), are the most abundant and genomically diverse entities within the coral holobiont [6–10]. In other holobionts, phages have been shown to modulate bacterial community composition [11–13] and defend against pathogens by adhering to mucosal surfaces and regulating bacterial colonization through lysis [14]. In corals, the pathogen *Vibrio coralliilyticus* can weaponize the lytic cycle of prophages in its competitors by triggering induction, gaining a competitive advantage over other symbiotic bacteria [15]. Shifts in phage community composition have also been observed in association with coral disease. For example, bleached and white plague–diseased tissues harbor distinct phage communities [16] and T4-like phages have been associated with black band disease mats [17], yet it remains unclear whether they play a causative role or if their compositional shifts reflect a generalized holobiont response to disease. At the reef scale, phages play a crucial role in shaping coral ecological interactions, including competitive dynamics for benthic space [18] and biogeochemical cycling [19].

Despite these advances, technical limitations in obtaining high-quality viral genomes from corals prevent the determination of phage contributions to holobiont functional diversity [20–22]. Coral host and Symbiodiniaceae genomes overwhelmingly dominate shotgun metagenomes, preventing appropriate coverage of viral sequences necessary for assembly. Moreover, viruses have high sequence diversity, lack hallmark genes, and display high levels of recombination, further complicating the assembly and characterization of viral metagenome–assembled genomes (vMAGs) [10, 23, 24]. Combined with the limited availability of reference viral genomes, most studies to date have relied on the analysis of read data without assembly of viral genomes [10, 22]. These studies also characterized the viral community using a taxonomy framework that uses phage tail morphology (former families *Myoviridae*, *Podoviridae*, and *Siphoviridae*) and the sequence identity of genes in these families to classify unknown viruses. This classification framework was abolished by the International Committee on Taxonomy of Viruses (ICTV) in 2022 in favor of a taxonomy system based on genomic composition, which better captures the evolutionary histories of viral groups [25]. Therefore, an updated perspective on the diversity and functional genomics of coral-associated viruses in light of current approaches and taxonomic framework is due.

Here, we introduce a size fractionation method for enriching viruses and bacteria in coral-associated metagenomes. We combine 33 metagenomes generated with this approach with publicly available datasets in a meta-analysis totaling 710 coral metagenomes, viromes, and bacterial isolates to reveal the genomic repertoire of coral holobiont viruses, focusing on phages. From this robust dataset, we identified 20,397 viral genomic sequences (2,121 vMAGs and 18,098 viral contigs) from 31 coral species distributed across seven oceanographic regions. The phage community was dominated by the class *Caudoviricetes* (tailed phages, realm *Duplodnaviria*) but also included *Monodnaviria* and *Varidnaviria* phages. The ability to assemble viral and bacterial genomes enabled the matching of phage–host pairs in coral microbiomes via CRISPR spacers. Phages most often infected hosts in the *Alphaproteobacteria*, *Gammaproteobacteria*, and *Bacteroidia* classes and encoded 109 unique metabolic genes and 64 unique bacteria–eukaryote interaction genes with the potential to affect bacterial metabolism and symbiotic interactions. These results transform our knowledge of the coral virome by identifying specific bacteria–phage–gene links involved in quorum sensing, sulfur cycling, DNA methylation, and molecular interactions with eukaryotic cells within the holobiont.

## Materials and methods

### Development of a viral and bacterial enrichment method

Fragments of the coral *Orbicella faveolata* (*N* = 28) were collected via SCUBA in July 2021, along the southwestern coast of Curaçao in the Caribbean (Table S1). Specimens of ~1 cm^3^ (containing mucus, tissue, and skeleton) were collected using a chisel and hammer and placed in Ziplock polyethylene bags with ambient seawater. The samples were placed on ice for an average of 20 min during transfer to the laboratory at the CARMABI research station, where ambient seawater was removed, and coral samples were flash frozen and stored at −80°C until later processing. The samples were subjected to two DNA extraction protocols for comparison: 19 samples were processed using the viral and bacterial enrichment (VBE) method developed here and 6 underwent bulk extraction and sequencing and were treated as controls. For both extraction methods, coral fragments were thawed, crushed to a fine gravel texture using a sterile mortar and pestle, and suspended in 150 μl of sterile artificial seawater. Control coral homogenates were extracted using a DNeasy PowerSoil Kit (QIAGEN, Germantown, MD), modified with the addition of 20 μl of proteinase K incubated at 56°C for 10 min prior to the kit’s lysis steps. For VBE, the coral homogenate was placed into a tube containing 0.2 g of 425- to 600-μm sterile glass beads (Sigma-Aldrich, St. Louis, MO) and vortexed (VWR Analog Vortex Mixer; VWR, Radnor, PA) at speed 3 (~ 600 rpm) for 5 min to disrupt the larger coral and algal symbiont cells. The supernatant was transferred to a clean tube, brought up to 1 ml with sterile ASW, and treated with DNAse I (20 U/ml final concentration) in DNAse I Buffer (Invitrogen, Waltham, MA) at 25°C for 2 h to degrade the coral host and algal DNA released in the previous step. DNAse activity was stopped with ethylenediaminetetraacetic acid (5 mM final concentration; Genesee Scientific, San Diego, CA), and the sample was filtered through an 8.0-μm membrane (Cytiva, Marlborough, MA). This filter size was intended to allow passage of the bacteria and viruses, while further removing the larger coral and Symbiodiniaceae cells. The flowthrough was collected, transferred to a 100 kDa Amicon Centrifugal Unit (Sigma-Aldrich, St. Louis, MO), and centrifuged at 3,200 *g* for 30 min to concentrate the bacteria and viruses together. After concentration, each side of the Amicon filter was rinsed and incubated at 56°C for 1 h (per side) in 200 μl Buffer T1 and 20 μl proteinase K from the NucleoSpin Tissue Kit (Macherey-Nagel Inc., Allentown, PA) and DNA extraction proceeded from Step 3 of the kit. DNA was eluted in 100 μl of PCR-grade water and quantified with a Qubit 2.0 Fluorometer (Invitrogen, Waltham, MA). A step-by-step description of the VBE protocol is publicly available on protocols.io [26]. Library preparation and whole-genome sequencing were conducted by Azenta Life Sciences (South Plainfield, NJ). Metagenomic libraries were generated with the NEBNext Ultra DNA Library Preparation kit following the manufacturer’s instructions (New England Biolabs, Ipswich, MA) and paired-end sequenced (2 × 150 bp) on a HiSeq 4000 platform (Illumina, San Diego, CA).

### Quantification of viral and bacterial enrichment in coral metagenomes

Raw metagenomic reads were adapter-trimmed, quality-filtered (trimq = 30, maq = 30), and entropy-filtered (entropy = 0.90) using BBDuk [27], generating 594,487,714 quality-controlled (QC) reads. To identify coral host and Symbiodiniaceae reads, the QC reads were mapped to the genome of the coral host, *O. faveolata* (GCA_001896105.1), and a representative Symbiodiniaceae genome (*Symbiodinium* sp. clade A Y106; GCA_003297005.1) using Bowtie2 (--mp 4 and -X 1000) [28]. Mapped reads were removed using SAMtools v1.18 [29] and quantified with SeqKit [30], yielding 336,288,370 coral and Symbiodiniaceae-filtered reads (Table S1). The QC reads and coral and symbiont-filtered reads from each sample were assembled separately using metaSPAdes v3.15.5 with default parameters [31]. Bacterial reads were identified with Kaiju v1.9.0 in each group using the proGenomes v3 database [32] and normalized by either the number of QC reads or coral and symbiont-filtered reads in each sample to obtain the percentage of bacterial reads in each metagenome. Bacterial metagenome-assembled genomes (bMAGs) were generated by integrating the single-sample coverage binning outputs from MaxBin2 v2.2.7 [33], MetaBat2 v2.15 [34], and CONCOCT v1.1.0 [35]. The resulting bins were consolidated and improved with the metaWRAP v1.2.1 [36] bin refinement module. The quality of refined bins was assessed with CheckM2 v1.0.2 [37] to select bins with ≥50% completion and ≤10% contamination. Viral identification and quantification were carried out using VIBRANT v1.2.1, which compared genes from all assembled contigs to the Kyoto Encyclopedia of Genes and Genomes (KEGG) KoFam, Pfam (v32), and Virus Orthologous Groups (VOG) [38]. Viral contigs were pooled by DNA extraction method to create two databases, “VBE” and “Control.” The number of reads mapped to their respective databases at 80% identity with SMALT v0.7.6 [39] was normalized by the total number of QC and filtered reads [40]. A summary of the bioinformatic pipeline is shown in Fig. S1. Comparisons between VBE and controls were based on Student’s *t*-test between the two groups, apart from bMAG assembly, which was compared based on a simple count of bMAGs generated from each group. Taxonomic assignment of phages was performed with the Phage Taxonomy Tool (PTT) [41], with the reference taxonomy edited to reflect updated ICTV classifications [42].

### Virus identification in global coral datasets

We assembled the global coral virus database (GCVDB) by combining viruses identified in the 28 Curaçao samples described above, 5 additional *O. faveolata* samples from Miami, FL (processed with VBE), and publicly available metagenomes (*N* = 355), viromes (*N* = 12), and genomes of bacteria (*N* = 310) isolated from reef-building corals (Tables S2 and S3 list the metadata and accession numbers for each dataset). Search terms and combinations of search terms including but not limited to “coral,” “metagenome,” “virome,” and “bacterial isolates” were used to identify publications and their associated datasets through Google Scholar, the National Center for Biotechnology Information (NCBI), the European Nucleotide Archive, and the JGI IMG/M Database [43]. The inclusion of samples was limited to coral metagenomes or bacterial genomes of isolates from corals of the order *Scleractinia* collected *in situ* (excluding aquaria and surrounding seawater) and to sequences with associated metadata describing the geographic location of sampling, the taxonomy of the coral host, and the methods used to collect and process samples. Metagenomic samples were restricted to Illumina sequences and excluded early 454 and Sanger sequencing studies.

The quality of the 310 bacterial genomes was assessed using CheckM v.2.0.12 [44]. VIBRANT identified 637 putative viral genomes in these bacterial genomes. After dereplication with Virathon [45], 178 representative viruses were added to the GCVDB. Metagenomes and viromes contained 36,006,392,282 raw metagenomic reads that were QC and assembled with the same parameters described above. No coral or symbiont read filtering was performed on this larger dataset, as not all coral species had available reference genomes. Contigs longer than 1,000 bp were screened by VIBRANT, and the identified viral genomic sequences were grouped by project, location, and host coral species, and co-binned into vMAGs with vRhyme v1.1.0 using the “longest” method, which dereplicates scaffolds, keeping the longest representative sequence [46]. Contigs within bins were subjected to further dereplication using Virathon and then N-linked with 1,000 Ns per link. N-linkages between contigs of a vMAG only indicate that contigs belong to the same vMAG, a step which is necessary for downstream dereplication of viral bins and quality assessment and do not indicate length [46]. N-linked vMAGs were dereplicated with Virathon, and their quality was assessed with CheckV v1.0.1, which estimates completeness and contamination based on the presence of hallmark genes by comparison with VOG, IMG/VR, RVDB, KEGG Orthology, PfamA, PfamB, and TIGRFAM [47]. This process generated 2,121 vMAGs and 18,098 viral contigs that were added to the GCVDB. Combined with the 178 viral contigs derived from bacterial isolates, a total of 20,397 unique putative viral genomes from coral holobionts compose the GCVDB (Table S4).

### Diversity of coral-associated viruses

Viral contigs annotated as “complete circular,” “high-quality draft,” and “medium-quality draft” by VIBRANT (*N* = 317) were combined with vMAGs annotated as “high-quality” and “medium-quality” by CheckV (*N* = 528) for further analyses (Tables S5 and S6). These genomes were grouped with the NCBI Viral RefSeq v1.1 database (accessed: 13 July 2023), which was first dereplicated using MIUViG-recommended parameters (95% average nucleotide identity, 85% alignment fraction) adopted by the Genomic Standards Consortium [48]. A comparison of genome relatedness between the GCVDB viruses and the RefSeq viruses was performed using GL-UVAB v0.6.pl, which calculates an all-versus-all Dice distance matrix based on the number of shared protein-encoding genes and their identity level [49]. The distance matrix was used to build a neighbor-joining tree containing 845 GCVDB viruses and 257 NCBI viruses. The tree was visualized and annotated using Interactive Tree of Life [50]. Taxonomic assignment of GCVDB viruses was performed by the PTT, which exclusively identifies prokaryotic viruses through protein similarity [41]. The “*PTT_virus_taxonomy.tsv*” reference data sheet was manually edited to reflect updated ICTV classifications [42]. Adonis and beta-dispersion analysis were performed with the R package Vegan [51].

### Bacteriophage host identification

Bacterial MAGs were generated from all metagenomic samples using the methods described above. Briefly, single-sample coverage binning outputs from MaxBin2 v2.2.7 [33], MetaBat2 v2.15 [34], and CONCOCT v1.1.0 [35] were consolidated and improved with metaWRAP v1.2.1 [36], resulting in 910 bins. CheckM2 v1.0.2 [37] was used to select bins with ≥50% completion and ≤10% contamination. Bins with low confidence predictions were removed (*N* = 6), resulting in 316 bMAGs taxonomically classified with GTDB-Tk v2.3.2 [52] (Table S7). To predict virus–host pairs, we employed a combination of provirus detection and CRISPR spacer matching (Table S8). A database of CRISPR spacers from the 316 bMAGs and 310 bacterial isolates was generated with minCED v0.4.3 [53], a tool derived from CRISPR Recognition Tools (CRT) v1.2 [54]. The resulting CRISPR spacer database was subsequently used to identify sequence homology matches with GCVDB using nucleotide BLAST (BLASTn). Sixty-three high-confidence pairs were generated using thresholds of ≤2 mismatches/gaps, 100% coverage to the spacer, and a sequence length of ≥20 nucleotides. Provirus detection was accomplished by mapping the GCVDB viruses to bMAGs with Minimap2 v2.24-r1122 [55]. Only the bMAG contigs that contained matches of 100% identity (no gaps) along the entire mapping length (*N* = 448) were selected to be assessed with CheckV, which identified 59 proviruses with host flanking regions. Viral contigs identified in bacterial isolate genomes by VIBRANT were categorized as proviruses (*N* = 177).

### Phage genetic repertoire

The genomes of the GCVDB viruses from metagenomic samples were compared to viruses identified in coral reef seawater metagenomes from the island of Curaçao [56]. These seawater metagenomes are paired with the Curaçao coral metagenomes described above (Table S1). Metabolic genes from viruses identified in seawater are shown in Table S9. We also searched for viral genes involved in bacteria–eukaryote interactions by a protein BLAST (BLASTp) comparison with a curated database of genes that have been experimentally shown to mediate host interactions in bacterial pathogens [57]. Because many of these genes have also been shown to be involved in commensal or mutualistic interactions in other bacterial species [58], here we refer to these genes as bacteria–host interaction genes or symbiosis genes. We used an e-value cut-off of ≤0.00001 and ≥40% identity across ≥20 amino acids to identify conserved domains in the amino acid sequences [59] (Table S10). For multiple quality hits on overlapping regions, the best hit was selected based on the e-value. Only metabolic genes identified in genomes with taxonomic annotations by PTT were included in the analysis of phage–bacteria interactions. Simpson’s Index of Diversity and Pielou’s evenness were used to compare the diversity and evenness of the gene distributions in seawater and corals. GCVDB phage genomes with host linkages and genes of interest (metabolism or symbiosis) were plotted with the R package genoPlotR [60].

### Tripartite network

A tripartite network of bacteria–phage–gene linkages was constructed using Cytoscape v3.9.1 [61]. To identify keystone viruses in this network, we treated shared metabolic and symbiosis genes as linkages between viruses. Keystones were identified using values of node degree (k; number of interactions), closeness centrality (cc; distance to all other nodes), and clustering coefficient (clust; connectivity of nearest neighbors) calculated by Cytoscape’s NetworkAnalyzer feature. We ranked each virus by the values of each of these three properties and calculated an average rank across the three to identify keystone viruses [62]. A Sankey diagram depicting the frequency of linkages between bacterial classes and viral families, as well as the frequency of metabolic and symbiosis genes encoded by the viral families, was generated using Google Charts.

## Results

### Virus and bacteria enrichment increases the recovery of microbial DNA in metagenomes

**Figure 1:**
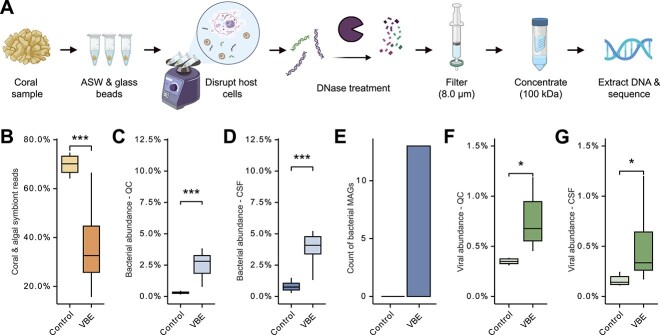
Enrichment of viruses and bacteria from coral samples. (A) Simplified workflow depicting the VBE method for enrichment of viruses and bacteria in coral metagenomes. (B–G) Recovery of coral and symbiont, bacteria, and viruses in VBE and control metagenomes. (B) Percent of coral and symbiont reads, (C) bacterial abundance within QC reads, (D) bacterial abundance within coral and symbiont-filtered (CSF) reads, (E) abundance of bMAGs from QC reads, (F) viral fractional abundance in QC reads and (G) in CSF reads. Stats represent *P* values from statistical tests: Student’s *t*-test in panels B, C, D, F, and G (^*^P < .05, ^*^^*^P < .01, ^*^^*^^*^P < .001). Workflow created with BioRender.com.

The VBE (Fig. 1A) metagenomes had 36.39 ± 0.10% (mean, SE) coral or symbiont reads, compared to 69.83 ± 0.13% (mean, SE) in the controls, a 52% reduction in the VBE metagenomes (*t*-test, *t*(23) = −5.58, *P* = 1.124e-05; Fig. 1B). Among quality-controlled reads, 2.72 ± 0.33% (mean, SE) of VBE reads were bacterial, compared to only 0.30 ± 0.03% (mean, SE; here and hereafter) in controls, a 9.01× enrichment in VBE (*t*-test, *t*(23) = 4.12, *P* = 4.15e-04; Fig. 1C). For coral and symbiont-filtered reads, VBE increased bacterial recovery by 5.02× (4.06 ± 0.09% and 0.81 ± 0.06% for VBE and control, respectively; *t*-test, *t*(23) = 4.29, *P* = 2.73e-04; Fig. 1D). High-quality bMAGs could only be generated from VBE metagenomes, with 13 total bMAGs with ≥50% completion and ≤10% contamination identified (Fig. 1E). VBE generated 543 putative viral contigs, compared to 76 from controls, which represented 0.74 ± 0.05% and 0.40 ± 0.05% of QC reads, respectively, a 1.87× increase for VBE (*t*-test, *t*(23) = 3.45, *P* = 2.17e-03; Fig. 1F). Among coral and symbiont-filtered reads, 0.46 ± 0.06% were viral in VBE and 0.16 ± 0.02% in controls, a 2.83× increase in viral recovery by VBE (*t*-test, *t*(23) = 2.6878, *P* = 1.31e-02; Fig. 1G).

### Updated taxonomic profile of coral holobiont phages

**Figure 2:**
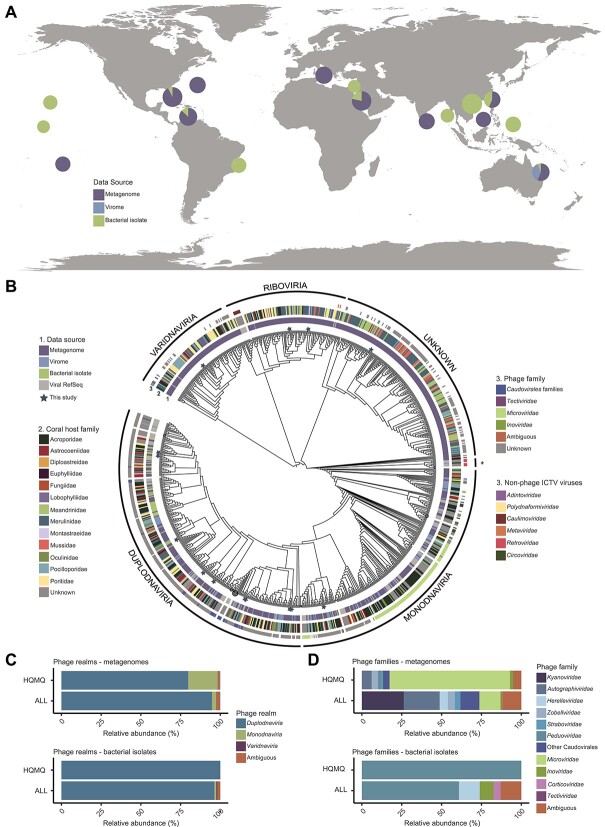
Coral virome diversity. (A) Distribution of sample locations and data types across ocean regions. Samples were grouped by oceanographic region for each pie chart. The radius of each pie chart represents the number of samples with log_10_(X + 4) transformation. The figure key indicates the type of dataset: metagenome, metavirome, or complete/draft bacterial genomes. (B) Proteomic tree of genomes and genome fragments in the GCVDB and their closest relatives from the NCBI viral RefSeq. The innermost ring (1) describes the data source (metagenome, virome, bacterial isolate), the second ring (2) depicts the family-level taxonomy of the coral host where the virus was identified, and the outermost ring (3) describes the family-level taxonomy of the viral genome (PTT taxonomy for phages and ICTV classification for RefSeq viruses). Viral realms were defined in the tree based on the position of RefSeq viruses. The branch labeled with an asterisk (^*^) contains *Riboviria* viruses. Branch lengths were omitted to better display the tree topology. Branches with three or more RefSeq members were collapsed (circles proportionally sized to the number of collapsed nodes). Stars indicate 14 viruses obtained by the VBE method. (C, D) Taxonomic classification of phages in the complete GCVDB (ALL) and in the subset of this database containing viral genomes of high or medium quality (HQMQ) at the realm (C) and family (D) taxonomic levels.

The 710 publicly available metagenomes, viromes, and bacterial isolates from 31 coral species and 7 ocean regions (Fig. 2A) analyzed here yielded 20,397 viral genomic sequences (18,098 vContigs and 2,121 vMAGs). These viral genomes were combined into the GCVDB, and hereafter, we refer to these genomic sequences as viruses for simplicity. A total of 846 viruses were classified as medium-quality, high-quality, or complete circular genomes (hereafter, high- and medium-quality viruses). A proteomic tree displaying the relationships between high- and medium-quality viruses in the GCVDB (*N* = 846) and viruses in the ICTV database (*N* = 99) shows GCVDB viruses spanning four viral realms according to the current ICTV classification (Fig. 2B). These include *Duplodnaviria* and *Varidnaviria* double-stranded DNA (dsDNA) viruses, *Monodnaviria* single-stranded DNA (ssDNA) viruses, and *Riboviria* RNA viruses similar to retroviruses with a DNA phase. Several branches of ICTV viral families that infect eukaryotes, including *Adintoviridae*, *Polydnaviriformidae*, *Caulimoviridae*, *Metaviridae*, and *Retroviridae*, included representatives from GCVDB. Bacteriophages, specifically, belonged to 3 realms and 25 families spanning both dsDNA and ssDNA viruses (Table S11).

The majority (68.95%) of viruses across the GCVDB were not taxonomically classified due to the low similarity with reference viruses. Among the high- and medium-quality viruses, most were identified as phages (58.85% and 97.96% in metagenomes and bacterial isolate genomes, respectively). Of the phages with taxonomic annotations, *Duplodnaviria* was the dominant realm regardless of quality (Fig. 2C). In metagenomes, this was followed by *Monodnaviria*, which represented 2.67% of the classified phages in the full GCVDB and 16.20% of the high- and medium-quality phages. In the genomes of bacterial isolates, *Monodnaviria* were absent among high and medium quality but comprised 1.24% when including low quality. 46.55% of *Duplodnaviria* viruses in the high- and medium-quality metagenomes did not have family classification under current taxonomy, resulting in an overrepresentation of ssDNA *Monodnaviria* families in Fig. 2D. Although these *Monodnaviria* families, *Microviridae* and *Inoviridae*, were the most abundant families identified among the high- and medium-quality metagenomes, dsDNA tailed phages belonging to the class *Caudoviricetes* were dominant in all other groups. Some dsDNA families annotated as *Duplodnaviria* were more closely related to viruses from other realms in the proteomic tree. For example, viruses classified as *Straboviridae* and *Ackermannviridae* fell within the *Varidnaviria*, and some *Peduoviridae* and *Ackermannviridae* were grouped within the realm *Monodnaviria*, which may result from the presence of shared genes between these groups.

We estimated the effects of coral host and ocean region in shaping virome composition by using the Jaccard beta diversity index. Viromes were significantly different across coral host families (*adonis*, *R*^2^ = 0.43, *P* = .001) and ocean regions (*adonis*, *R*^2^ = 0.36, *P* = .001). However, the project and subproject that generated the data explained the most variation in Jaccard’s beta diversity (60.78% and 71.99%, respectively). As any given project was from a single ocean region, and each subproject includes both a single ocean region and a single coral species, these variables are not independent, and the effects of biology and geography cannot be disentangled. The data displayed different levels of dispersion by ocean region (*betadisper*, *F* = 4.4, *P* = 2.86e-04), coral host family (*betadisper*, *F* = 15.7, *P* = <2.2e-16), project (*betadisper*, *F* = 18.3, *P* = < 2.2e-16), and subproject (*betadisper*, *F* = 7.2, *P* = <2.2e-16), indicating that the Adonis PERMANOVA results are affected by nonhomogeneous dispersion of the data.

### Coral phages preferentially infect *Alphaproteobacteria*, *Bacteroidia*, and *Halanaerobiia*

**Figure 3:**
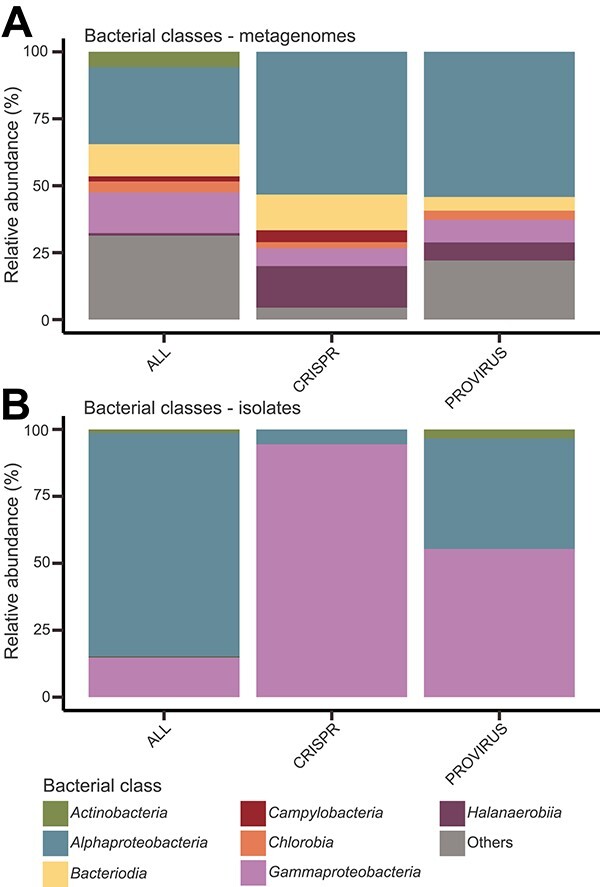
Bacteriophage–bacteria interactions. Frequency of (A) bMAGs and (B) isolates in the metagenomic dataset (ALL) and within those with CRISPR spacer or prophage linkages, at the taxonomic level of class. Classes with abundance <2% (except for *Halanaerobiia*, which represented <1% of bMAGs, but 7%–16% of linkages) were grouped as “others”.

To link viruses with their hosts, we binned and taxonomically classified 316 bMAGs from the metagenomes (belonging to 41 bacterial classes) and combined them with the 310 publicly available bacterial isolate genomes from coral (5 classes) for a total of 626 putative bacterial hosts. We identified 299 putative links between 144 hosts and 275 GCVDB viruses based on the presence of proviruses (*N* = 236) and CRISPR spacer matches (*N* = 63) (Table S8). 24.2% of bacterial isolates (*N* = 75) and 15.5% of bMAGs (*N* = 49) contained at least one provirus. *Alphaproteobacteria*, the most abundant bMAG class (28.80%), was overrepresented among phage-linked bMAGs (53.33% of CRISPR spacer links and 54.24% of provirus links; Fig. 3A). *Gammaproteobacteria* were the second most abundant class of bMAGs representing 15.19% but were underrepresented in their CRISPR spacer (6.67%) and provirus (8.47%) links. The classes *Halanaerobiia* and *Bacteroidia*, both comprising obligate anaerobes, represented <13% of the bMAGs combined, yet had over twice as many CRISPR spacer linkages as *Gammaproteobacteria* bMAGs. Among bacterial isolates, however, *Alphaproteobacteria* links were underrepresented, accounting for only 5.56% of CRISPR spacer links and 41.24% of provirus links, and *Gammaproteobacteria* isolates were overrepresented in their linkages, accounting for 94.44% of CRISPR spacer linkages and 55.37% of provirus linkages (Fig. 3B). We also identified viruses predicted to infect the putative coral mutualist *Endozoicomonas*, which harbored more than one prophage per genome (Table S8).

### Metabolic and eukaryote interaction genes differ between coral and seawater viruses

**Figure 4:**
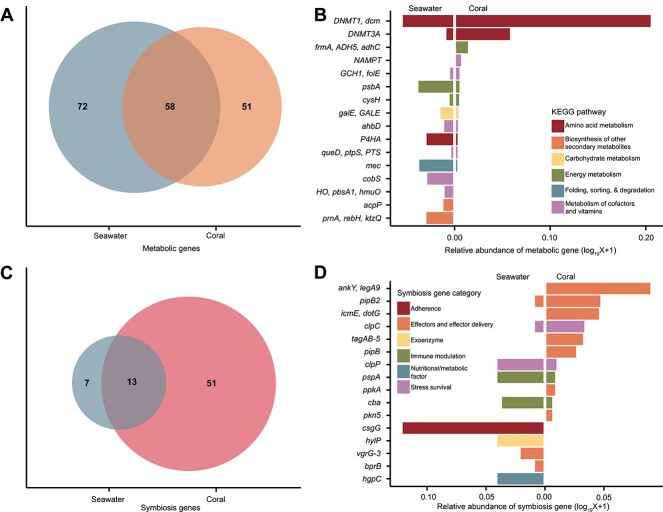
Genomic repertoire of coral-associated bacteriophages. (A) Count of unique metabolic genes (defined based on KEGG Orthologs) identified in coral and seawater bacteriophages. An overlap indicates those identified in both sample types. (B) Frequencies of the most common metabolic genes, calculated as the sum of viruses encoding each gene within each sample type and sorted by abundance. The colors indicate the KEGG pathways to which those genes belong. (C) Count of unique symbiosis genes identified in coral and seawater bacteriophages. (D) Frequencies of the 10 most common symbiosis genes, calculated as the sum of the phages containing each gene within each sample type and sorted by the frequency in corals.

The 3,912 seawater and 6,173 coral viruses classified here as phages encoded 181 unique genes with KEGG ortholog annotations (11.3% of coral and 27.3% of seawater phages). Seawater viruses encoded more unique metabolic genes (*N* = 72) than corals (*N* = 51), despite a >22-fold increase in sampling effort (18 seawater metagenomes were used here for comparison versus 400 coral metagenomes; Fig. 4A). Among the 58 metabolic genes shared between coral and seawater viruses, the most common were involved in amino acid metabolism, energy metabolism (photosynthesis), and sulfur relay (Fig. 4B). Only 5 of the 10 most common metabolic genes in coral phages were also among the 10 most common in seawater phages. Metabolic gene α-diversity, as expressed by Simpson’s index of diversity, was higher in seawater (*D* = 0.95) than in corals (*D* = 0.61). Additionally, metabolic genes were more evenly distributed in seawater than in coral phages (E_Pielou_ = 0.72 in seawater and E_Pielou_ = 0.41 in corals), where a single gene, DNA cytosine-5 methyltransferase 1 (*DNMT1, dcm*), accounted for 60.35% of all metabolic genes identified. *DNMT1* was also the most abundant gene in seawater, yet only accounted for 13.25% of the metabolic genes. Together, the four most abundant metabolic genes in coral viruses, *DNMT1, dcm*; DNA cytosine-5 methyltransferase 3A (*DNMT3a*; 14.32%); *S*-(hydroxymethyl)glutathione dehydrogenase/alcohol dehydrogenase (*frmA*, *ADH5*, *adhC*; 3.29%), and nicotinamide phosphoribosyltransferase (*NAMPT*; 1.57%), accounted for 80% of the metabolic genes encoded by viruses in corals, whereas in seawater, 21 genes constitute that same 80% threshold. These common metabolic genes in coral phages are involved in DNA methylation (*DNMT1, dcm; DNMT3a*), the oxidation of long-chain alcohols and formaldehyde in several metabolic pathways (*frmA, ADH5, adhC*), and the metabolism of cofactors and vitamins (nicotinate and nicotinamide metabolism; *NAMPT*).

Coral and seawater phages also encoded 71 unique genes classified here as eukaryote interaction genes. These genes were previously classified as virulence factors based on their role in known pathogens [57,58], but many have roles in bacteria–eukaryotic interactions in commensal or mutualistic relationships [58] and were referred to here as eukaryote interaction or symbiosis genes. Fifty-one of these genes were unique to coral phages, 13 were shared between corals and seawater, and only 7 were unique to the seawater phages (Fig. 4C). The majority (70.00%) of the genes in coral phages encoded proteins with ankyrin domains involved in bacteria–eukaryote commensal and mutualistic interactions. This includes the most abundant gene, *ankY/legA9*, encoding an ankyrin motif-containing protein and accounting for 22.86% of the symbiosis gene group in coral phages (Fig. 4D). They were followed by genes involved in stress survival (10.29%) and immune modulation (9.14%). Coral phages carried a variety of other genes, such as *pipB2* (T2SS effector; 11.42%) and *icmE* (DotG T4SS central channel protein; 11.14%), which are components of effector delivery systems that may be involved in the direct molecular interaction between bacteria and eukaryotes. Seawater phages not only primarily encoded genes involved in adherence (35.92%, with *csgG*, involved in curli production, as the most abundant [32.04%]) and immune modulation (19.42%) but also encoded genes related to stress survival at a similar frequency as in coral viruses (11.65%).

**Figure 5:**
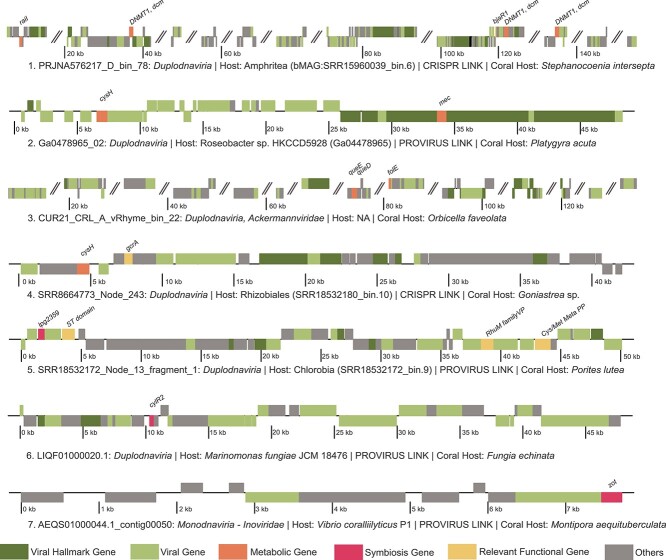
Genome plots of GCVDB viruses. Genome annotations of seven viral genomes of interest due to their host link or the presence of metabolic or symbiosis genes. The colors indicate gene classifications, and angled parallel lines indicate separations between contigs within viral MAGs.

We selected seven high-quality viral genomes encoding genes with putative roles in host metabolism and symbioses to explore in detail (Table S12), six of which have been linked to bacterial hosts (Fig. 5). Among the *Duplodnaviria* viruses, PRJNA576217_D_bin_78 (genome 1) was linked via CRISPR spacer to a bMAG classified as *Amphritea* sp. (SRR15960039_bin.6), family *Oceanospirillaceae* of *Gammaproteobacteria*. This virus encoded a DNA cytosine-5 methyltransferase 1 (*DNMT1, dcm*), an acyl-homoserine lactone (AHL) synthase (*raiI*), and a quorum-sensing system regulator (*bjaR1*). Another *Duplodnaviria*, Ga0478965_02 (genome 2), was integrated into the genome of *Roseobacter* sp. HKCCD5928 (*Alphaproteobacteria*) isolated from the coral *Platygyra acuta*. This virus encoded a phosphoadenosine phosphosulfate reductase (*cysH)* and a CysO sulfur-carrier protein-S-L-cysteine hydrolase (*mec*). A representative of *Ackermannviridae* (CUR21_CRL_A_vRhyme_bin_22; genome 3) was not linked to a host but encoded queuosine biosynthesis genes typically involved in the phage–host evolutionary arms race. *queD*, encoding a 6-pyruvoyltetrahydropterin/6-carboxytetrahydropterin synthase; *queE*, a 7-carboxy-7-deazaguanine synthase; and *GCH1*, *folE*, a GTP cyclohydrolase, all play roles in deazaguanine DNA modifications in phages. SRR8664773_Node_243 (genome 4) was CRISPR spacer linked to a *Rhizobiales* bMAG (SRR18532180_bin.10) from a *Goniastrea minuta* metagenome and encoded the metabolic gene (*cysH*) as well as bacterial cell cycle regulator (*gcrA*). SRR18532172_Node_13_fragment_1 (genome 5), a *Duplodnaviria* representative derived from *Porites lutea* skeleton, was integrated into a bMAG (SRR18532172_bin.9) from the same sample. This virus encoded a sulfotransferase domain (ST domain), cysteine and methionine metabolism PLP-dependent enzyme (Cys/Met Meta PP) as well as virulence factors, including a type IV secretion system effector (*lpg2359*) and a RhuM family virulence protein (RhuM family VP). *Duplodnaviria* virus LIQF01000020.1 (Genome 6) was identified in the genome of *Marinomonas fungiae* JCM18476 isolated from the coral host *Fungia echinata* and encoded the virulence factor *cylR2*, a cytolysin regulator. The isolated coral pathogen *V. coralliilyticus* strain P1 carried an integrated *Monodnaviria* virus in the *Inoviridae* family of chronic filamentous viruses (AEQS01000044.1_contig00050; Genome 7) encoding the gene for the zonula occludens toxin (*zot*).

### Tripartite network

**Figure 6:**
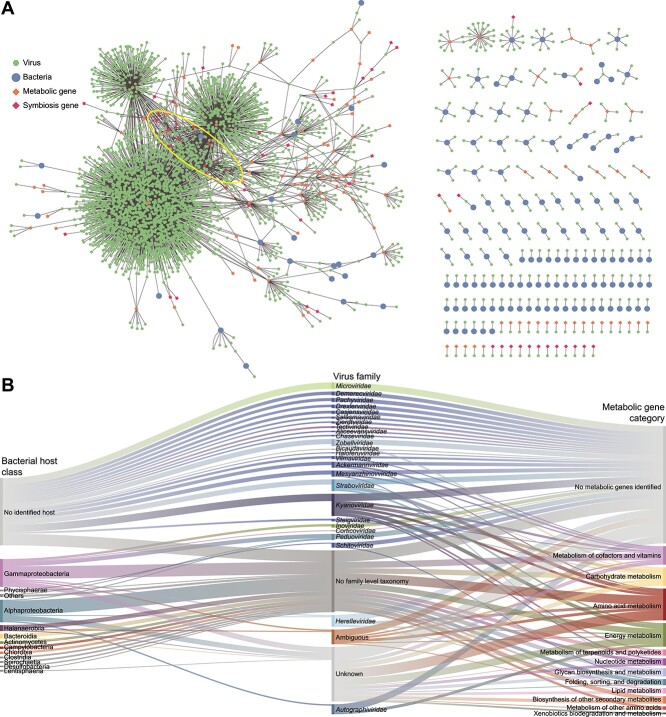
Bacteria–phage–gene network. (A) GCVDB viruses were connected to bacterial hosts (isolate and bMAG) according to the host prediction described in the methods and to metabolic and symbiosis genes according to their functional annotations. The ellipse indicates the position of viruses identified as keystones in the network analyses. (B) Alluvial plot displays abundant bacterial classes (left) linked to viral families (center) found to interact with them though CRISPR spacer and provirus linkages identified in this study. On the right, viral families are linked to metabolic genes encoded in their genomes.

Links between viruses and their putative hosts (*N* = 299) and the metabolic and symbiosis genes encoded by viruses in the GCVDB (*N* = 5,219) were used to construct a network where shared genes were the edges connecting phages in addition to phage–bacteria connections via CRISPR and prophage links in a bipartite network (Fig. 6A displays a version of this network where genes are shown as nodes for visualization purposes). Among bacteria, the average clustering coefficient, which describes the connections between neighbors of a node, was zero. Thus, in the case where multiple viruses were linked to the same host, these viruses did not share metabolic or virulence genes. The top six ranks of keystone viruses based on shared metabolic and symbiosis genes included 17 viruses (Table S13), all of which were classified as *Duplodnaviria* without family-level classification or host linkages, highlighting our lack of information about these important viruses. These viruses primarily encoded the most common genes within the GCVDB, such as *DNMT1, dcm*, *DNMT3A*, involved in DNA methylation, and *ankY/legA9*, involved in bacteria–eukaryote interactions. To better visualize the distribution of the host–phage–gene connections, we displayed these links in an alluvial plot with phages grouped at the family level and hosts at the class level (Fig. 6B). This plot shows the high representation of genes involved in amino acid and energy metabolism (important for phage particle production during infection) and other gene functions related to cofactors and vitamins and carbohydrate metabolisms, encoded by viruses belonging to several families of tailed bacteriophages (*Autographiviridae*, *Herelleviridae*, *Kyanoviridae*, and *Straboviridae*) infecting *Alphaproteobacteria*, *Gammaproteobacteria*, and *Halanaerobia*, among others.

## Discussion

Here, we introduce a size fractionation method to increase the recovery of viral and bacterial DNA in coral metagenomes. By combining these metagenomes with data generated across coral metagenomic and culture-based studies worldwide, including 710 coral metagenomes, viromes, and bacterial isolates, we identified 531 high- and medium-quality metagenome-assembled viral genomes from corals. These viruses infect diverse hosts and encode genes that contribute to several holobiont functions interactions, specifically in phage defense mechanisms and bacteria–coral symbioses.

### Enrichment of viruses and bacteria in coral metagenomes

VBE captured high- and medium-quality viral genomes across four of the six viral realms recognized by the ICTV, except for *Adnaviria* and *Ribozyviria* (Fig. 2B). By using size fractionation and sequencing viruses and bacteria together, the VBE method incurred fewer compositional biases compared to previous methods [63]. Chloroform treatment used in previous studies efficiently removes bacteria but selects against enveloped viruses, some nonenveloped viruses, and a third of tailed phages [63–65]. Cesium chloride (CsCl) gradients enhance viral recovery [63] but severely bias viral diversity due to differences in viral capsid density [66] and often require the use of amplification methods that incur further biases [67]. In viral studies that avoided these biases, viral genomes were either not assembled, limiting the analyses to single reads [10] or focused primarily on viruses of eukaryotes [68]. Here, bacterial and viral genome recovery through VBE was possible even at a relatively low-sequencing coverage (37 million reads and 5.5 billion bases per sample on average). This recovery represents an improvement from previous studies that required coverages from 58 to 146 million reads [69–72] to assemble bMAGs and were most successful with samples devoid of coral tissue, such as in studies focused on the skeleton communities [69]. The differences observed in the outputs between VBE and control samples could be partially attributed to differences in sequencing depth, as VBE sample coverage was, by chance, 40% higher than the control samples. However, this difference was not proportional to the 9 times increase in bacterial recovery (800% increase) and 2 times increase in viral recovery (100% increase). VBE uses a coral fragment not much larger than a parrotfish bite, which is minimally invasive to corals. Therefore, this method could be easily adapted for the recovery of bacterial and viral RNA [73], which would presumably require larger amounts of input material. This method also pools all holobiont compartments (skeleton, tissue, and mucus), and adaptations are required for application to specific compartments [70, 74]. Increasing the size of the coral input sample and sequencing depth and incorporating long-read data may lead to the resolution of more complete genomes covering a larger diversity.

A potential caveat of the symbiont filtering approach used here to calculate the efficacy of the VBE method after filtering eukaryotic reads bioinformatically is that reads were mapped to a single Symbiodiniaceae genome at a low-sequence identity threshold with the goal of removing sequences from Symbiodiniaceae or related genera. To test the effect of this approach, we mapped our reads to the genomes of four Symbiodiniaceae genera common in *O. faveolata* in the Caribbean: *Symbiodinium*, *Breviolum*, *Cladocopium*, and *Durusdinium* [75]*.* This increased the number of reads mapped to Symbiodiniaceae from 36% to 44% in the VBE and from 70% to 75% in the control. However, the difference between VBE and control changed only marginally, from a 52% reduction to 58%, confirming that the initial approach removed most Symbiodiniaceae reads. As a result, the enrichment of bacterial reads using VBE was 5.0 times in the older symbiont filtering versus 4.5 times in the filtering with four genera, and for viruses, it changed from 2.8 to 2.5 times. These differences were small, and the statistical difference between VBE and control was maintained.

### Updated taxonomy of coral-associated viruses

The discontinuation of morphology-based viral taxonomic assignments by the ICTV in 2022 has implications for studying bacterial viruses in corals. This taxonomic reorganization included the creation or relocation of 1 order, 22 families, 30 subfamilies, 321 genera, and 862 species while simultaneously eliminating families such as *Podoviridae*, *Siphoviridae*, and *Myoviridae*, along with the order *Caudovirales* [42]. In previous studies, these families were consistently the most abundant members of the coral holobiont [9, 10, 20]. Within the GCVDB, we identified viruses in the realms *Duplodnaviria*, *Monodnaviria*, *Varidnaviria*, and *Riboviria*. Among the phages, the vast majority belonged to the class *Caudoviricetes*, which includes all tailed bacterial and archaeal viruses with icosahedral capsids and a dsDNA genome [42]. The most abundant families under the new taxonomy were *Kyanoviridae and Autographiviridae*, which represented 65.0% of *Caudoviricetes*. The family *Kyanoviridae* likely represents many of the since-abolished *Myoviridae* phages previously identified in corals. These “T4-like” phages share the myovirus morphotype and current representatives in this family exclusively infect *Cyanobacteria* [42]. The *Kyanoviridae* phages in our dataset encoded many metabolic and symbiosis genes but did not have host linkages, preventing a better understanding of their roles in corals. This lack of linkages could be due to a lack of integration capability, infection of hosts that do not employ CRISPR-Cas defense systems, or low-abundance and genetically diverse hosts for which metagenome-assembled genomes (MAGs) were not assembled [48, 56]. *Autographiviridae* viruses include cultured representatives that typically exhibit a lytic lifestyle and podophage morphology and primarily infect *Gammaproteobacteria* hosts [76]. This contrasts with *Autographiviridae* in our dataset, as two were linked with *Alphaproteobacteria* hosts.

### Coral viruses encode a limited but distinct repertoire of metabolic genes

Through the expression of metabolic genes during infection, phages can impact the function and ecological relationships of their hosts [77, 78]. Despite a massively higher sampling effort of corals (*N* = 400) in comparison with seawater (*N* = 18), coral phages had a lower frequency and a less diverse repertoire of metabolic genes, primarily encoding genes involved in amino acid metabolism. These results suggest a strong selection of few metabolic genes in phage genomes associated with corals. Differences in sequencing coverage were likely not an explanation for this pattern, as 6,173 viral genomes were recovered from corals versus 3912 from seawater. Yet, coral–viral genomes were less complete (16.03% average completeness) compared to seawater (51.72%), which could contribute to this difference in metabolic gene recovery. Nevertheless, this completeness estimate relies on hallmark genes and might be biased against novel viruses, particularly those from understudied systems such as coral [47]. The genes encoding DNA (cytosine-5)-methyltransferase 1 (*DNMT1, dcm*) and DNA (cytosine-5)-methyltransferase 3A (*DNMT3A*), comprising 60.40% of the metabolic genes in coral viruses, are involved in DNA methylation for evasion of restriction-mediated resistance against phage infection [79]. They were found in viruses infecting eight different bacterial families, indicating a widespread evolutionary arms race between coral viruses and their bacterial hosts [80]. Another possible function of *DNMT1, dcm* is in the colonization of mucosal surfaces. In Group B *Streptococcus* specifically, knockout of *dcm* was found to reduce binding to immobilized mucin [81]. We predict that in corals, these genes are involved in the evasion of bacterial defense during lytic infections and possibly in enhancing bacterial host mucus colonization.

*S*-(hydroxymethyl) glutathione dehydrogenase (*frmA, ADH5, adhC*), the next most frequent metabolic gene in coral viruses, plays a role in formaldehyde metabolism, which could be important for biomass and energy production of methylotrophs and other fermenting bacteria [82]. Corals display significant diurnal oxygen fluctuations, with daytime photosynthetic oxygen production followed by rapid consumption by heterotrophic metabolism at night and resultant shifts to anaerobic metabolism [83], which may explain the presence of formaldehyde metabolism genes in coral-associated phages. Viruses carrying *frmA, ADH5, adhC* genes, if infecting anaerobes that proliferate during nocturnal respiration, may bolster downstream production of formate, a key electron donor in anaerobic respiration [84]. The contribution of fermentation to coral holobiont metabolism during nighttime oxygen depletion and the phage contribution to this shift should be the focus of future studies.

### Genes for bacteria–eukaryote interactions are rare in coral–phage genomes

Previous studies indicated that ~40% of viral sequences from corals were related to pathogen–host interactions, annotated as “Virulence, Disease, and Defense” [10]. Although these genes are involved in the virulence of known pathogens, many of the same genes can be involved in commensal or mutualistic bacteria–eukaryote interactions in nonpathogens, showing that they mediate bacteria–host interactions across the full spectrum of symbiosis [58]. Therefore, we refer to this group of genes as symbiosis genes or eukaryote interaction genes. Here, 3.3% of phage genomes in the GCVDB and 2.2% of phage genomes in the seawater samples encoded at least one symbiosis gene. This frequency is close to that observed in coral–seawater boundary layer viruses [57], where 2%–4% of the viral community encoded these genes, an order of magnitude lower than the frequency observed in other coral-associated viruses [10]. This difference may be due to the use of a read-based bioinformatic approach that may recruit bacterial reads and inflate the estimates in the study that found higher frequencies [10], compared to the assembly and MAG-binning approach used here. The distributions and functions of these symbiosis genes also differed between studies. Here, seawater phages often encoded genes related to adherence and invasion, consistent with coral boundary layer samples [57]. In contrast, most symbiosis genes encoded by coral viruses were ankyrin motif-containing proteins (*ankY/legA9*) that mediate protein–protein interactions between hosts and symbionts [85, 86]. Ankyrin motif-containing proteins transferred by phages can enhance bacteria–host symbioses through the suppression of host immune cells [85, 87]. In other bacterial hosts, these proteins represent a family of Type IV secretion system effectors [88]. The similarly high frequency of Icm/Dot type IV secretion system central channel proteins (*icmE/dotG*) among coral viruses indicates an important role of effector delivery systems in phage–bacteria–coral interactions, enabling bacteria to directly translocate effectors into prokaryotic competitor cells and eukaryotic host cells through an injection apparatus [89, 90]. Greater microbe–microbe competition in the dense coral microbiome [91] and frequent bacteria–animal interactions may drive the selection of these secretion systems and ankyrin motif-containing proteins in the phages identified here.

### Host links and network

Associating uncultivated viruses with their microbial hosts remains a challenge not only for coral microbiomes but also for any phage–host studies [48, 56]. Here, our ability to identify bMAGs enabled predictions of virus–host pairs through CRISPR matches and integrated proviruses. The most abundant bMAG hosts, *Alphaproteobacteria* and *Gammaproteobacteria*, displayed an interesting trend where *Alphaproteobacteria* hosts were overrepresented among bMAGs with linkages and *Gammaproteobacteria* hosts were underrepresented, suggesting higher frequency of viral infection in the more abundant *Alphaproteobacteria*, consistent with observations of *Alphaproteobacteria* in coral reef seawater [56]. Lower completeness of gammaproteobacterial genomes (~7% less complete than *Alphaproteobacteria* bMAGs, *t*-test, *t*(137) = 2.51, *P* = 1.34e-02) could partially but not fully explain the underrepresentation of *Gammaproteobacteria* linkages. This may simply indicate a lower frequency of viral infections of *Gammaproteobacteria* in corals for unknown reasons. *Halanaerobiia* and *Bacteroidia* constituted a minor fraction of bMAGs, yet they represented a substantial percentage of the phage linkages. This observation aligns with a previous investigation in coastal seawater environments, wherein an abundance of free rRNA, indicative of recently lysed cells, was noted for copiotrophic and low-abundance bacteria [92]. Here, bMAGs of these bacterial groups had multiple phage linkages, suggesting that these taxa are susceptible to infection by multiple viruses.

**Figure 7:**
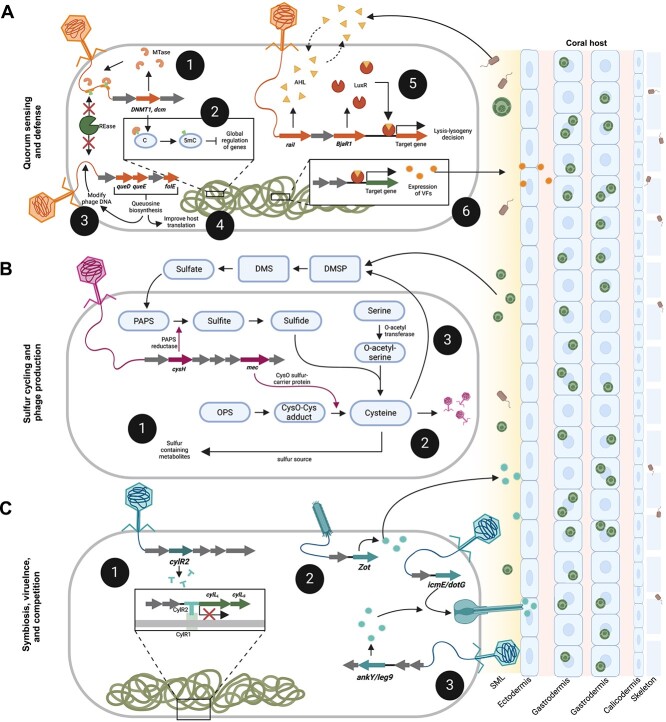
Conceptual hypotheses for mechanisms underpinning phage roles on coral-associated microbiomes. (A) Viral infection and gene transfer impact bacterial quorum sensing and defense where phage-encoded methyltransferases (1) protect phage nucleic acids from host-encoded restriction endonucleases and (2) regulate adhesins and metabolic factors with putative roles in carbohydrate metabolism and mucin adherence. Genes involved in the synthesis of queuosine precursors may be used to (3) modify phage DNA as protection against host restriction systems or (4) increase queuosine levels in host tRNAs, improving the host’s translational efficiency. Quorum-sensing genes encoded by phages may influence (5) lysis–lysogeny decisions and (6) the expression of genes related to eukaryotic–host interactions (virulence genes). (B) Phages may contribute to sulfur cycling through (1) impacting production of sulfur-containing metabolites that are effective against oxidative stress, (2) the diversion of sulfur toward cysteine production and assimilation into phage proteins, and (3) interference in the synthesis of DMSP due to the deviation of the DMSP precursor cysteine toward phage proteins. (C) Phages may impact virulence and competition within the coral holobiont by (1) regulating the expression of bacterial cytolysins, (2) expression of exotoxins that increase the permeability of coral host tissues, and (3) encoding type IV secretions system central channel proteins and effectors that can mediate bacteria–bacteria or bacteria–eukaryote interactions. Figure created with BioRender.com.

A closer inspection of viral genomes with connections with hosts and genes of interest revealed the genomic underpinnings of phage–bacteria–coral interactions. As quantifying viral abundances across datasets produced with different methods was not viable, these genomes were selected based on their high estimated genome quality, in addition to the presence of genes of interest and a host link, to identify possible mechanisms of virus–host interactions based on their high-confidence predictions. *Duplodnaviria* phage PRJNA576217_D_bin_78 encodes an AHL synthase (*raiI*) and a LuxR family transcriptional regulator (*bjaR1)*, which are both involved in quorum sensing. RaiI produces AHL, which, upon reaching a critical threshold within a bacterial cell, triggers AHL-signaling and the initiation of the expression of virulence genes [93]. BjaR1 acts as a quorum-sensing transcriptional regulator involved in the response to AHL [94]. The presence of both genes in the same phage suggests that this phage coopts the AHL signaling of its bacterial host (bMAG SRR15960039_Bin.6) to modulate gene expression and/or direct lysis–lysogeny decisions. The host, *Amphritea sp.*, is an *Oceanospirillaceae* in the *Gammaproteobacteria* class [95]. A bMAG of the same genus was found across metagenomes of a stony coral tissue loss diseased *Stephanocoenia intersepta* [70] and has been found in association with black band disease [96]. We speculate that the virus identified here may be involved in the pathogenicity of *Amphritea* in corals through modulation of quorum-sensing signaling (Fig. 7A).

Evidence of the phage–host evolutionary arms race was present in several genomes, as many viruses encoded DNA methyltransferases likely for evasion of restriction-mediated resistance. Another mechanism of this restriction evasion was demonstrated in the genome CUR21_CRL_A_vRhyme_bin_22 (Genome 3 in Fig. 5). The queuosine biosynthesis genes encoded by this phage, *queD*, *queE*, and *folE*, are widespread in phages, especially in the genomes of those with pathogenic hosts [97]. These genes are suggested to modify phage DNA as protection against host restriction systems and contribute to the level of queuosine in host tRNAs, improving host translational efficiency [97].

*Roseobacters* constitute a significant portion of the coral mucous microbiome, and although the precise nature of their interactions with coral remains ambiguous, there is evidence suggesting their potential roles as probiotics in coral reproduction and defense [98]. The degradation and production of dimethylsulfoniopropionate/dimethylsulfide (DMSP/DMS) by *Roseobacters* is considered a nutrient source for corals [99, 100]. Here, a phage genome (Ga0478965_02) within a *Roseobacter* isolate (sp. HKCCD5928) encoded metabolic genes related to sulfur metabolism, specifically assimilatory sulfate reduction (phosphoadenosine phosphosulfate reductase; *cysH*) and the transfer of sulfur during cysteine production (CysO sulfur-carrier protein; *mec*). Viruses commonly utilize sulfur metabolism genes to increase the rates of cysteine production for viral particle assembly [41]. Yet, these sulfur genes were encoded in a prophage, Ga0478965_02. It is possible that these genes are transcribed only when the prophage enters the lytic cycle [101]. Alternatively, its expression during lysogeny may contribute to the degradation of DMSP and production of sulfur-containing metabolites by the *Roseobacter* host (Fig. 7B). Other bacterial hosts associated with the skeletal components of coral hosts exhibited connections to viruses that carried multiple genes associated with the metabolism of sulfur-containing amino acids and metabolites, like SRR8664773_Node_243 and SRR18532172_Node_13_fragment_1. Although these phage-encoded genes could potentially play a role in DMSP cycling and phage production, the production of sulfur-containing metabolites has also been linked to resistance against oxidative and nitrosative stress in various bacterial hosts [102].

Genes related to effector delivery systems, with potential roles in bacterial competition and interactions with the coral host, were common symbiosis genes in the GCVDB. Often, these phage-encoded effector proteins are multifunctional, but often, they convert bacterial hosts from nonpathogenic to virulent [103] (Fig. 7C). One of the viral genomes identified here, *Duplodnaviria* virus LIQF01000020.1, encoded genes involved in the regulation of cytolysin, an exotoxin that lyses prokaryotic and eukaryotic cells [104]. A prophage-encoding zonula occludens toxin (Zot) previously detected in the coral pathogen *V. coralliilyticus* strain P1 was also observed here. These observations support the idea that certain coral diseases may result from lysogenic conversion [105, 106]. However, some of these virulence genes can also be involved in nonpathogenic functions in the molecular interactions between bacteria and eukaryotic cells when they are called “fitness factors” [58]. Here, these cells may include the coral animal cells, the endosymbiotic algae, or other eukaryotes in the holobiont.

The network analysis revealed several keystone *Duplodnaviria* viruses based on their shared metabolic genes, which makes them more connected within the network and more able to diversify the functional capacity of bacteria within the holobiont. Low clustering across bacterial members of the network shows that the viruses infecting the same bacterial host do not share metabolic genes. This indicates that different viruses create distinct virocell metabolism upon infection of the same host [107] and supports the idea of viruses as a reservoir of genetic information that can be horizontally acquired by bacteria under changing conditions.

The results described here reveal the basis of mechanisms by which bacteriophages interact with bacteria within the coral holobiont, with potential downstream effects on bacteria–bacteria and bacteria–coral interactions. The large dataset and high resolution gained from the assembly and binning of genomes enabled the use of stringent quality thresholds, increasing confidence in the annotations and predictions made. The gene-resolved phage–host network highlighted phages’ selective repertoire of metabolic genes that can impact bacterial communication, competition, and molecular interactions with the coral host, illuminating the genomic underpinnings of phage–bacteria–coral tripartite symbioses.

## Supplementary Material

Wallace2024_TableS1_wrae132

Wallace2024_TableS2_wrae132

Wallace2024_TableS3_wrae132

Wallace2024_TableS4_wrae132

Wallace2024_TableS5_wrae132

Wallace2024_TableS6_wrae132

Wallace2024_TableS7_wrae132

Wallace2024_TableS8_wrae132

Wallace2024_TableS9_wrae132

Wallace2024_TableS10_wrae132

Wallace2024_TableS11_wrae132

Wallace2024_TableS12_wrae132

Wallace2024_TableS13_wrae132

Wallace_2024_FigS1_resub_wrae132

Wallace_2024_supplementary_legends_6_28_24_BW_wrae132

## Data Availability

Coral metagenomic sequence data generated for this project are available on NCBI (PRJNA1061506 and PRJNA975592). The code used to conduct this analysis is publicly available on GitHub (https://github.com/Silveira-Lab/Wallace_Coral_Holobiont_Viruses). Assembled viral genomes are available on Figshare: https://doi.org/10.6084/m9.figshare.24968805.v1. Viral genomes with multiple contigs are N-linked. Bacterial MAGs are available on Figshare: https://doi.org/10.6084/m9.figshare.25000160.v1.
