# Supplementary figures and images for: Globally distributed bacteriophage genomes reveal mechanisms of tripartite phage–bacteria–coral interactions
Source: ISME J. 2024 Jul 20;18(1):wrae132. doi: 10.1093/ismejo/wrae132 (PMC11309003; doi:10.1093/ismejo/wrae132)

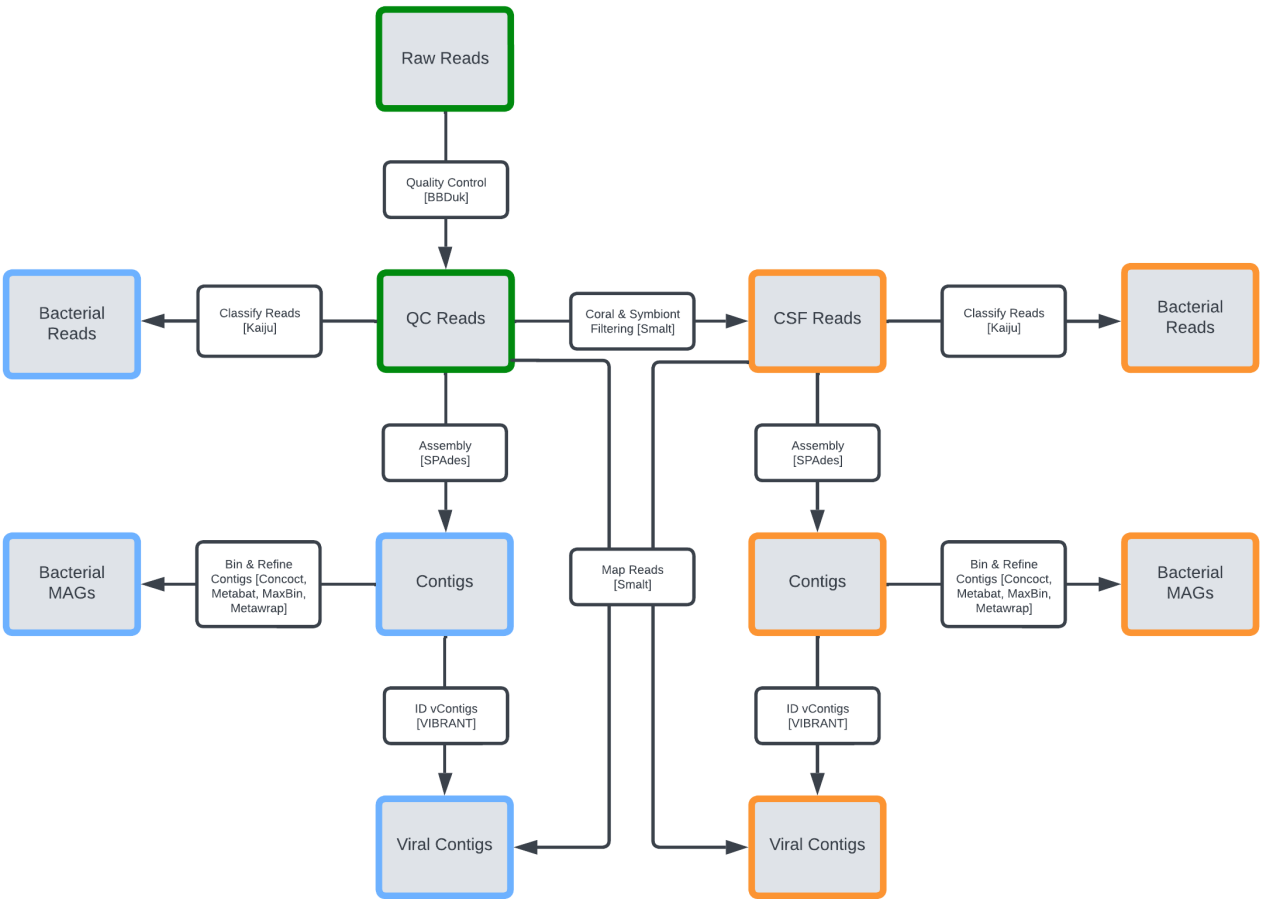

Supplement: Wallace_2024_FigS1_resub_wrae132 [file wallace_2024_figs1_resub_wrae132.pdf]
