# Supplementary material for: Globally distributed bacteriophage genomes reveal mechanisms of tripartite phage–bacteria–coral interactions
Source: ISME J. 2024 Jul 20;18(1):wrae132. doi: 10.1093/ismejo/wrae132 (PMC11309003; doi:10.1093/ismejo/wrae132)
Supplement: Wallace_2024_supplementary_legends_6_28_24_BW_wrae132 [file wallace_2024_supplementary_legends_6_28_24_bw_wrae132.docx]

**Supplementary Figure Legends**

**Figure S1 | Summary of bioinformatic pipeline for analysis of viral and bacterial enrichment method.** Workflow diagram depicts the steps of bioinformatic analysis from raw reads and quality control steps, to host filtering, read classification, bMAG assembly, and the identification and quantification of viral reads.

**Supplementary Table Legends**

**Table S1 | Curaçao 2021 coral sample metadata.** Metadata for the coral-associated metagenomes used in development of the viral and bacterial enrichment method including the collection date, sampling location, sample type, coral host species, method used, and sequence read data after each stage of bioinformatic processing. Coral and symbiont filtered reads are termed CSF reads and quality-controlled reads are termed QC reads.

**Table S2 | Bacterial isolate metadata.** Metadata for the genomes of coral-associated bacterial isolates analyzed in this study include the sample and file names, bacterial taxonomic classification, coral host of isolation, geographic location, and accession numbers. References and links to original studies were included if available.

**Table S3 | Metagenome and virome metadata.** Metadata for the coral-associated metagenomes and viromes analyzed in this study include the sample and project names, accession numbers, sample type, coral host, notes on the methods/treatments used, collection date, depth, geographic location, and sequencing platform. Affiliations and links to original studies were included if available.

**Table S4 | Global coral virus database**. Description of genomes and genome fragments composing the global coral virus database (GCVDB), including the sample name, associated project, genome length, and taxonomy at the realm and family level.

**Table S5 | Viral contig quality summary**. Quality summary of viral contigs in the GCVDB including the contig name, data source, scaffold name, type of virus, and quality as determined by VIBRANT.

**Table S6 | Viral MAG quality summary**. Quality summary of viral MAGs in the GCVDB including the contig name and length, provirus status and length, gene count, viral gene count, host gene count, CheckV quality, MIUViG quality, completeness, completeness method, contamination, kmer frequency, and warnings. Contig length includes N-linkages for vMAGs.

**Table S7 | Bacterial MAGs.** Description of each bacterial metagenome assembled genome (bMAGs) binned in this study, filtered to include only those with ≥ 50% completion and ≤ 10% contamination. The table includes CheckM2 quality scores, genome length, GC content, sample and project identifiers, and bacterial taxonomy as determined by GTDB-Tk v2.3.2.

**Table S8 | Phage-host linkages.** Description of phage-host linkages, including both bMAG and bacterial isolate hosts. Viruses are annotated at the realm and family level and the linkage method is indicated as CRISPR spacer or provirus.

**Table S9 | Metabolic genes identified in phages from coral and seawater metagenomes.** The table indicates the protein ID, scaffold ID, KEGG Orthology (KO), AMG annotation ID and name, Pfam annotation ID and name, as well as the sample type (seawater vs. coral) from which the viral genome was derived.

**Table S10 | Symbiosis genes identified in phages from coral and seawater metagenomes.**

The table indicates the query sequence ID (qseqid), subject sequence ID (sseqid), percent of identical matches (pident), length, mismatches, number of gap openings (gapopen), the start of alignment in query (qstart), the end of alignment in query (qend), the start of alignment in subject (sstart) the end of alignment in subject (send) the E-value (evalue), bitscore, and the group (seawater vs. coral) from which the virus was derived. These results were filtered to include an e-value cut-off of ≤ 0.00001 and ≥ 40% identity across ≥ 20 amino acids.

**Table S11 | Family-level taxonomic classification of GCVDB phages.** The genomes labeled as “unknown” were not able to be classified. “NA” was assigned to genomes classified as bacteriophages without a family level classification under current ICTV taxonomy. The data is presented as the number of genomes, the percentage of the total, and the percentage of the viruses identified as phages. Summaries are separated by the data source (metagenome or bacterial isolate).

**Table S12 | Gene annotations for viral genomes displayed in Figure 6.** Genome annotations for viruses include the protein ID, virus name, start and end position of each protein, strand, gene category, annotations and accession numbers, and for vMAGs, the old start and end positions of proteins within each separate contig. Contigs within vMAGs are separated by rows listed as “N-Link” in the accession column.

**Table S13 | Predicted keystoneness of GCVDB viruses.** Top six rankings among keystone viruses. Keystones were identified by a mean rank of the number of interactions (K), closeness centrality (cc), and clustering coefficient (clust) scores generated with Cytoscape v3.9.1. Data includes the virus quality, family- and realm-level taxonomy, genome length, and a count of the genes of interest (metabolic or virulence genes).
